# Segmental hypopigmented acneiform nevus with *FGFR2* gene mutation^⋆^

**DOI:** 10.1016/j.abd.2021.09.021

**Published:** 2023-05-12

**Authors:** Yongyi Xie, Baoyi Liu, Zhouwei Wu

**Affiliations:** a Department of Dermatology, Shanghai General Hospital, Shanghai, China; b Department of Dermatology, Shanghai Jiao Tong University School of Medicine, Shanghai, China

Dear Editor,

Segmental acneiform nevus associated with mutations of Fibroblast Growth Factor Receptor 2 (*FGFR2*) was first reported by Munro and Wilkie.^1^ Very few similar cases were reported under different names making this disorder confusing. Here, the authors report a new case with a missense mutation in the *FGFR2* gene and summarized the features of this specific entity.

A 13-year-old Chinese boy presented with a segmental hypopigmented patch on his left abdomen and back following the Blaschko line since birth. Since the age of 10, comedones, scattered red papules, pustules, and nodules developed on the hypopigmented patch (Fig. 1A-B). Dilated follicular ostia with keratin plug, less pigmented terminal hairs with abnormal curled growth pattern, and follicle-centered hypomelanosis were identified under the Dermoscopy examination (Fig. 1C). During early childhood the patient had a minor delay in mental development and an attention deficit disorder, but no solid evidence of mental deficiency was found when he presented. The patient’s general health status, magnetic resonance imaging scan, skeletal X-Ray, and routine laboratory examinations were normal. Their family history was unremarkable.

**Figure 1:**
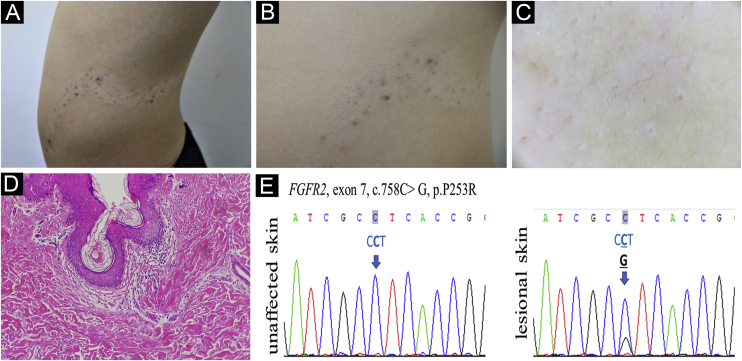
(A) Acneiform nevus on hypopigmentation patch following the Blaschko line. (B) Detailed comedones, inflammatory papules and nodules. (C) Follicular plug, curled hairs, and follicle-centered hypomelanosis by Dermoscopy, ×10. (D) Dilated plugged follicular infundibula and perifollicular lymphohistiocytic infiltrate (Hematoxylin & eosin , ×100. (E) *FGFR2* DNA sequence of the acneiform nevus and contralateral unaffected skin of the patient is shown. The heterozygous somatic mutation is indicated by the arrow

Venous blood was taken from the patient and his parents, and skin biopsies from the lesional and normal skin of the patient were performed. Histological examination showed dilated plugged follicular infundibula and perifollicular lymphohistiocytic infiltrate (Fig. 1D). Sanger sequencing of *FGFR2* (NM_022970) detected the heterozygous mutation c.758C>G in exon 7 in the affected skin (Fig. 1E) which was neither present in unaffected skin nor in the lymphocytes from him and his parents. The sequence alteration represents a somatic mosaic mutation leading to a Pro253Arg amino acid change (CCT253CGT).

Few similar cases have been reported under different names (Table 1). A patient with mosaic *FGFR2* mutation (p.Ser252Trp) showed similar clinical symptoms.^2^ Kiritsi et al.^3^ reported a severe case in a patient presenting with multiple segments involved. Ma et al.^4^ described a similar case but not confirmed by gene investigation. In addition, some documented nevus comedonicus cases with hypopigmentation may be the same disease.^5^

**Table 1:** Main features of published cases of a segmental acneiform nevus with hypopigmentation

Reference	Age at onset	Age at diagnosis	Sex	Distribution of lesions	Morphology of lesions	Mutation
1	12	14	Male	Single segmental (left arm)	Mostly comedones with scattered inflammatory papules	*FGFR2* mutation (p.Ser252Trp)
2	10	15	Male	Multiple segmental (right chest, trunk, shoulder, and arm)	Multiple comedones, inflammatory papules and pustules with hypopigmentation	*FGFR2* mutation (p.Ser252Trp)
3	1	12	Male	Multiple segmental (left and right sides of chest, trunk, shoulders, arms, and right face)	Multiple comedones, inflammatory papules, pustules, nodules, scars with hypopigmentation	*FGFR2* mutation (p.Pro253Arg)
Ours	1	13	Male	Single segmental (left abdomen and back)	Multiple comedones and inflammatory papules, pustules, nodules with hypopigmentation	*FGFR2* mutation (p.Pro253Arg)
4	1	25	Male	Single segmental (right chest and back)	Mostly comedones with scattered inflammatory papules with hypopigmentation	NA

Acneiform lesions are the primary feature of this entity. *FGFR2* mutation in keratinocytes could induce the hypercornification of the pilosebaceous duct and inflammatory response.^2^ The p.Pro253Arg mutation is located in the highly conserved linker region of *FGFR2* and leads to ligand-dependent *FGFR2* activation *in vivo* due to a conformational change that increases ligand-binding affinity.^3^ Extensive acneiform lesions, and depigmentation of hair, skin and eyes have been described in Apert syndrome in which two-thirds of patients exhibit a germline *FGFR2* mutation.^6^ The acneiform lesions responded to treatment with isotretinoin 20 mg daily like those in Apert syndrome.^3^, ^6^

The presence of early-onset hypopigmentation is another persistent characteristic feature reported in almost all published cases except the first case in which whether there was pigmentation change was not mentioned. It could be caused by the failure of melanosome transfer from melanocytes to keratinocytes or by elevated IL-1α-mediated postinflammatory hypopigmentation.^2^

Based on clinical and molecular data of these published cases, here, the authors propose that ‘segmental hypopigmented acneiform nevus with *FGFR2* mutation’ might be a more comprehensive description for this specific entity. In addition, the authors suggest any atypical nevus comedonicus with a feature of hypopigmentation and/or inflammatory lesion should consider this disorder and bear in mind that postzygotic mosaicism for a genetic disease such as Apert syndrome can also affect the gonads, thus resulting in a risk of transmission to offspring.^6^

## Financial support

None declared.

## Authors’ contributions

Yongyi Xie: Contribution with data collection and interpretation; preparation and writing of the manuscript; manuscript review.

Baoyi Liu: Contribution with data collection and interpretation; preparation and writing of the manuscript; manuscript review.

Zhouwei Wu: Approval of the final version of the manuscript; manuscript review.

## Conflicts of interest

None declared.
